# Smartphone Cardiac Rehabilitation, Assisted Self-Management Versus Usual Care: Protocol for a Multicenter Randomized Controlled Trial to Compare Effects and Costs Among People With Coronary Heart Disease

**DOI:** 10.2196/15022

**Published:** 2020-01-27

**Authors:** Jonathan Charles Rawstorn, Kylie Ball, Brian Oldenburg, Clara K Chow, Sarah A McNaughton, Karen Elaine Lamb, Lan Gao, Marj Moodie, John Amerena, Voltaire Nadurata, Christopher Neil, Stuart Cameron, Ralph Maddison

**Affiliations:** 1 Institute for Physical Activity and Nutrition Deakin University Geelong Australia; 2 Melbourne School of Population and Global Health University of Melbourne Parkville Australia; 3 Westmead Applied Research Centre, Faculty of Medicine and Health University of Sydney and Westmead Hospital Westmead Australia; 4 Deakin Health Economics Institute of Health Transformation Deakin University Geelong Australia; 5 Geelong Cardiology Research Unit Barwon Health Geelong Australia; 6 Department of Cardiology Bendigo Health Bendigo Australia; 7 Department of Medicine Western Health and University of Melbourne Sunshine Australia; 8 Applied Artificial Intelligence Institute Deakin University Geelong Australia

**Keywords:** telemedicine, telerehabilitation, mHealth, myocardial ischemia, coronary artery disease, exercise, behavioral medicine, health services accessibility, costs and cost analysis

## Abstract

**Background:**

Alternative evidence-based cardiac rehabilitation (CR) delivery models that overcome significant barriers to access and delivery are needed to address persistent low utilization. Models utilizing contemporary digital technologies could significantly improve reach and fidelity as complementary alternatives to traditional center-based programs.

**Objective:**

The aim of this study is to compare the effects and costs of the innovative *Smartphone Cardiac Rehabilitation, Assisted self-Management* (SCRAM) intervention with usual care CR.

**Methods:**

In this investigator-, assessor-, and statistician-blinded parallel 2-arm randomized controlled trial, 220 adults (18+ years) with coronary heart disease are being recruited from 3 hospitals in metropolitan and regional Victoria, Australia. Participants are randomized (1:1) to receive advice to engage with usual care CR or the SCRAM intervention. SCRAM is a 24-week dual-phase intervention that includes 12 weeks of real-time remote exercise supervision and coaching from exercise physiologists, which is followed by 12 weeks of data-driven nonreal-time remote coaching via telephone. Both intervention phases include evidence- and theory-based multifactorial behavior change support delivered via smartphone push notifications. Outcomes assessed at baseline, 12 weeks, and 24 weeks include maximal aerobic exercise capacity (primary outcome at 24 weeks), modifiable cardiovascular risk factors, exercise adherence, secondary prevention self-management behaviors, health-related quality of life, and adverse events. Economic and process evaluations will determine cost-effectiveness and participant perceptions of the treatment arms, respectively.

**Results:**

The trial was funded in November 2017 and received ethical approval in June 2018. Recruitment began in November 2018. As of September 2019, 54 participants have been randomized into the trial.

**Conclusions:**

The innovative multiphase SCRAM intervention delivers real-time remote exercise supervision and evidence-based self-management behavioral support to participants, regardless of their geographic proximity to traditional center-based CR facilities. Our trial will provide unique and valuable information about effects of SCRAM on outcomes associated with cardiac and all-cause mortality, as well as acceptability and cost-effectiveness. These findings will be important to inform health care providers about the potential for innovative program delivery models, such as SCRAM, to be implemented at scale, as a complement to existing CR programs. The inclusion of a cohort comprising metropolitan-, regional-, and rural-dwelling participants will help to understand the role of this delivery model across health care contexts with diverse needs.

**Trial Registration:**

Australian New Zealand Clinical Trials Registry (ACTRN): 12618001458224; anzctr.org.au/Trial/Registration/TrialReview.aspx?id=374508.

**International Registered Report Identifier (IRRID):**

DERR1-10.2196/15022

## Introduction

### Background

Cardiac rehabilitation (CR) is a cost-effective multifactorial intervention that plays a critical role in the secondary prevention of coronary heart disease (CHD) [1-4]; however, low rates of participation in traditional center-based programs (ie, face-to-face delivery) [5] continue to limit impact on individual, clinical, and economic outcomes. Uptake and adherence to center-based CR are influenced by diverse factors, but access barriers such as limited program availability, transport restrictions, conflicting domestic/occupational responsibilities, and geographic isolation are key contributors [6-9]. Establishing new programs in diverse locations could improve accessibility, but this may not be feasible, given the high infrastructure costs associated with center-based facilities [10,11]. The traditional CR paradigm cannot meet the needs of many eligible individuals, and complementary alternatives that deliver evidence-based support outside of face-to-face settings are urgently needed to complement current services.

Home-based delivery overcomes many access barriers and can have comparable short-term effects on mortality, exercise capacity, and health-related quality of life [12], but it often lacks oversight and guidance from CR professionals, which are central to best-practice center-based programs.

Rapid advances in mobile communications and wearable sensor technologies could bridge the gap between home- and center-based delivery models by combining near-universal accessibility with responsive individualized clinical oversight. CR delivered via landline telephone, SMS, and Web-based mechanisms has demonstrated promising potential [13]. However, few interventions have incorporated emerging digital technologies that can support more comprehensive, responsive, and interactive intervention delivery that emulates center-based support [14]. We previously developed a 12-week exercise-based mobile health (mHealth) intervention, named Remote Exercise MOnitoring Trial for Exercise-based Cardiac Rehabilitation (REMOTE-CR), to address this unmet need [15]. Using mobile, Web, and wearable sensor technologies, our bespoke intervention remotely connected participants with exercise physiologists to receive real-time individualized supervision and coaching during exercise training from any geographic location with a local or mobile internet connection. In a noninferiority randomized controlled trial (RCT), we showed that the effect of REMOTE-CR on functional capacity was as good as traditional center-based programs. The trial also demonstrated comparable effects on modifiable cardiovascular risk factors and motivational outcomes, substantial program delivery cost savings via reduced infrastructure requirements, as well as high usability, acceptability, and end-user demand for REMOTE-CR as a usual care service [10,16]. Collectively, these findings suggest complementing existing center-based programs with effective mHealth delivery models could increase overall engagement with CR by allowing more individuals to access individualized intervention support.

However, REMOTE-CR trial findings also identified opportunities for further development. First, REMOTE-CR focused primarily on exercise training, whereas comprehensive CR should also incorporate health behavior change and education, management of other lifestyle risk factors—including healthy diet and tobacco smoking—and adherence to prescribed medications [2,3]. Second, the advantages of mHealth CR are likely to be greatest in regional and rural areas where access to existing services is lowest [17]; however, the predominantly urban sample in our trial meant we could not determine impact in regional or rural areas. Third, long-term maintenance of self-management behaviors is a key component of CR [2,3], and—in combination with substantial cost-efficiencies [10,18,19]—near-universal accessibility makes mHealth CR well suited for supporting long-term self-management. However, our REMOTE-CR intervention provided only 12 weeks of support. Fourth, our previous trial actively facilitated referral and priority enrollment into center-based rehabilitation for participants randomized to the control group. This differs from usual care practice as referral rates are far from optimal [20,21], and individuals are typically required to self-initiate engagement with usual care services.

We have subsequently developed a 24-week multifactorial intervention (*Smartphone Cardiac Rehabilitation, Assisted self-Management * [SCRAM]) that addresses previous participant feedback [16], includes more comprehensive multifactorial behavior change support, and integrates an additional 12-week maintenance phase to assist participants’ transition toward self-determined long-term adherence to self-management behaviors.

### Objectives

This protocol describes the rationale and design of a multicenter RCT that aims to compare the effects and costs of SCRAM on modifiable cardiovascular risk factors with usual care CR among urban-, regional-, and rural-dwelling individuals with CHD. Usual care CR was chosen as the comparator as it is the standard of care. We hypothesize that the effects of SCRAM on primary and secondary outcomes will be superior to usual care CR alone and that SCRAM will be cost-effective.

## Methods

### Design

**Figure 1:**
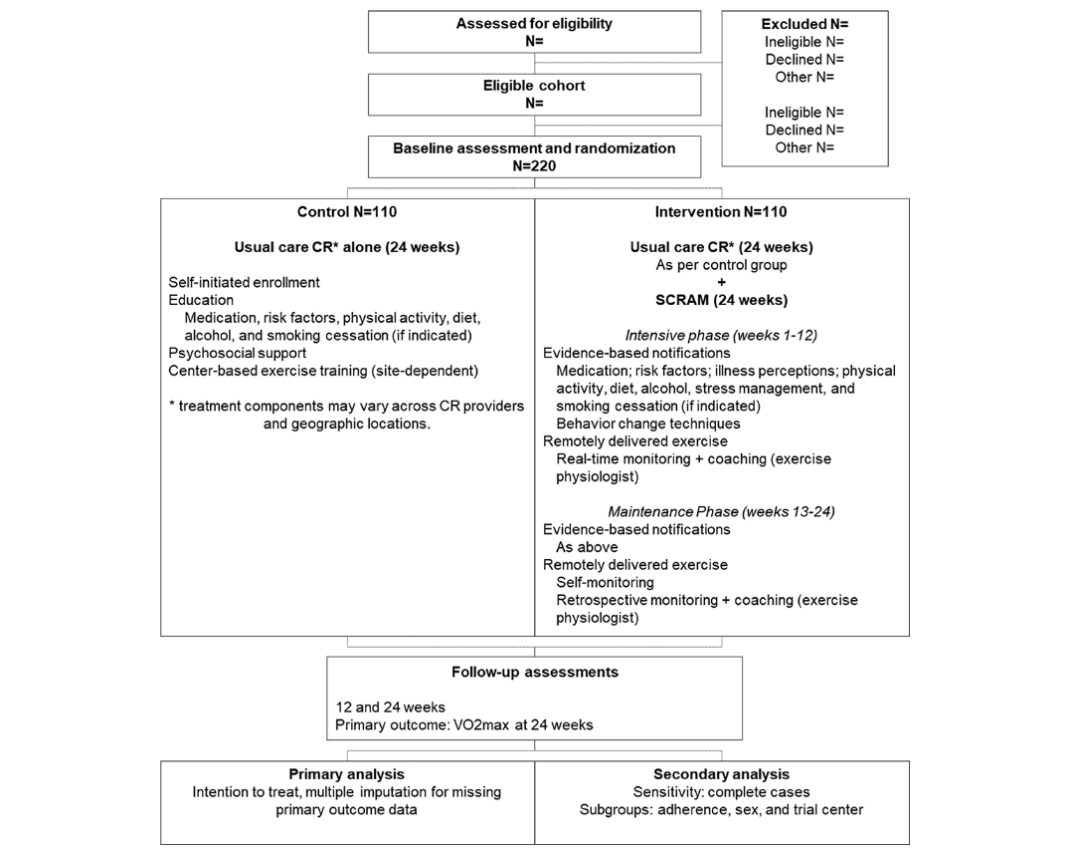
Consolidated Standards of Reporting Trials design schematic. CR: cardiac rehabilitation; SCRAM: Smartphone Cardiac Rehabilitation, Assisted self-Management.

A multicenter investigator- and assessor-, and statistician-blinded parallel 2-arm RCT is being conducted to compare effects of SCRAM and usual care CR on maximal aerobic exercise capacity, modifiable cardiovascular risk factors, and self-management behaviors (Figure 1). Process and economic evaluations aim to determine participants’ perceptions of the SCRAM and usual care programs and the cost-effectiveness of SCRAM, respectively.

The trial protocol was prospectively registered with the Australian New Zealand Clinical Trials Registry (ACTRN12618001458224) on August 30, 2018, and it adheres to the Standard Protocol Items: Recommendations for Interventional Trials 2013 statement [22]. The intervention has been described according to recommendations in the Template for Intervention Description and Replication and Consolidated Standards of Reporting Trials (CONSORT; electronic health [eHealth] extension) statements (Multimedia Appendices 1-3). Reporting of trial outcomes will adhere to the CONSORT statement and its eHealth extension [23-25].

### Setting

The trial is being conducted at 1 metropolitan (Sunshine Hospital, Western Health) and 2 regional (University Hospital Geelong, Barwon Health; Bendigo Hospital, Bendigo Health) health care providers in Victoria, Australia. These trial centers collectively serve approximately 1.5 million individuals and provide acute coronary care services to individuals living in large regional and rural catchment areas across Northern and Western Victoria. This enables sampling of a geographically diverse cohort that includes metropolitan-, regional-, and rural-dwelling participants, which is particularly important as age-standardized rates of CHD hospitalizations and access to traditional center-based CR are worse outside metropolitan areas in Australia [26,27].

We anticipate recruiting equal numbers of participants via each trial center; a priori prediction of the ratio of metropolitan, regional, and rural participants is not possible, but it will be reported together with trial outcomes.

### Recruitment

Recruitment began at all trial centers on November 12, 2018, and it is monitored via monthly reporting to the trial steering group, comprising the trial investigators. Research nurses identify eligible individuals before hospital discharge and via outpatient cardiac clinics, provide potential participants with trial information and consent forms, obtain consent to refer interested individuals to the research team, and document reasons for declining participation. A researcher subsequently confirms individuals’ interest in the trial, answers unresolved questions, and schedules baseline assessment appointments for those who volunteer to participate.

### Eligibility

Eligible participants are adults (18+ years) with recently diagnosed CHD (angina, myocardial infarction, and coronary revascularization within the previous 6 months), who are clinically stable outpatients (no CHD-related hospitalization within 6 weeks of baseline assessment) and can understand and write English. Participants are excluded if they have New York Heart Association class III/IV heart failure, terminal disease, significant non-CHD exercise limitations, or contraindications for maximal exercise testing. Participants with an implanted pacemaker or automated defibrillator are excluded because of wearable sensor manufacturer recommendations. Smartphone ownership is not required; participants are lent a smartphone for the duration of the intervention period if required. Participants who cannot complete baseline primary outcome assessment are ineligible for randomization, and participants who experience exercise-induced medical complications may be ineligible, pending referral for medical assessment and approval.

### Randomization

Upon completion of baseline data collection, participants are randomized at a 1:1 ratio to receive 24 weeks of usual care CR alone (control) or the 24-week SCRAM program (intervention). Treatment allocation follows a computer-generated schedule prepared by a biostatistician who is not involved in recruitment, treatment allocation, or outcome assessment; the randomization schedule is stratified by sex and trial center and uses random permuted blocks (n=2 and 4). A centralized Web-based randomization system (Research Electronic Data Capture [REDCap]; Vanderbilt University) ensures allocation concealment until the time of randomization. Access to the randomization schedule is restricted to the blinded trial biostatistician. The trial steering group is permitted access to summarized allocation progress data to monitor recruitment. Researchers undertaking data collection have no access to the randomization schedule other than to retrieve participants’ concealed treatment allocation at baseline assessments.

### Blinding

Primary outcome assessment will be blinded; participants are blinded at baseline (ie, prerandomization) but cannot be blinded thereafter because of the nature of the treatments. A trial investigator who is not involved with data collection may be unblinded to participants’ treatment allocation if it is medically necessary. The biostatistician will remain blinded to treatment allocation throughout the analysis of trial outcomes.

### Treatments

#### Usual Care Cardiac Rehabilitation

All participants retain access to standard outpatient CR (ie, usual care), regardless of treatment allocation. Usual care CR typically includes face-to-face delivery of support and education to adhere to medical treatment and health-promoting lifestyle behaviors, as well as supervised exercise training. Specific program components, content, frequency, and duration vary substantially across health care providers; however, the large majority of health care providers in Australia offer education and exercise components [28].

Usual care CR is not delivered as part of this trial; as per standard clinical practice, participants are required to self-initiate engagement by requesting referral from their local health care provider.

Varied usual care CR provision is expected because of the multisite trial design. Participants who receive acute treatment at the large recruiting hospitals but live in outlying regional and rural areas will receive usual care CR via smaller regional and rural health services. Given the number of CR programs across the collective trial catchment area, it is not possible to describe all candidate programs in detail, but stratification of the treatment allocation sequence by trial center will help to balance variation across treatment groups. Participants continue to take prescribed medications and medical treatments throughout the trial period.

#### Control

Participants randomized to the control arm are advised to seek referral to usual care CR—as described above—if they wish to participate. No additional support is provided (Figure 1).

#### Intervention

Participants randomized to the intervention arm receive the 24-week SCRAM intervention. SCRAM is a multicomponent dual-phase intervention that provides participants with a comprehensive, individualized, evidence-based program of exercise training and modular behavioral self-management support via a bespoke mHealth platform. An initial 12-week intensive phase supports the uptake of exercise and CHD self-management behaviors. A subsequent 12-week maintenance phase provides lower-intensity support to help participants transition toward autonomous, long-term adherence beyond the intervention period (Figure 1). The 24-week duration is considered appropriate to observe sustained behavior change [29].

**Figure 2:**
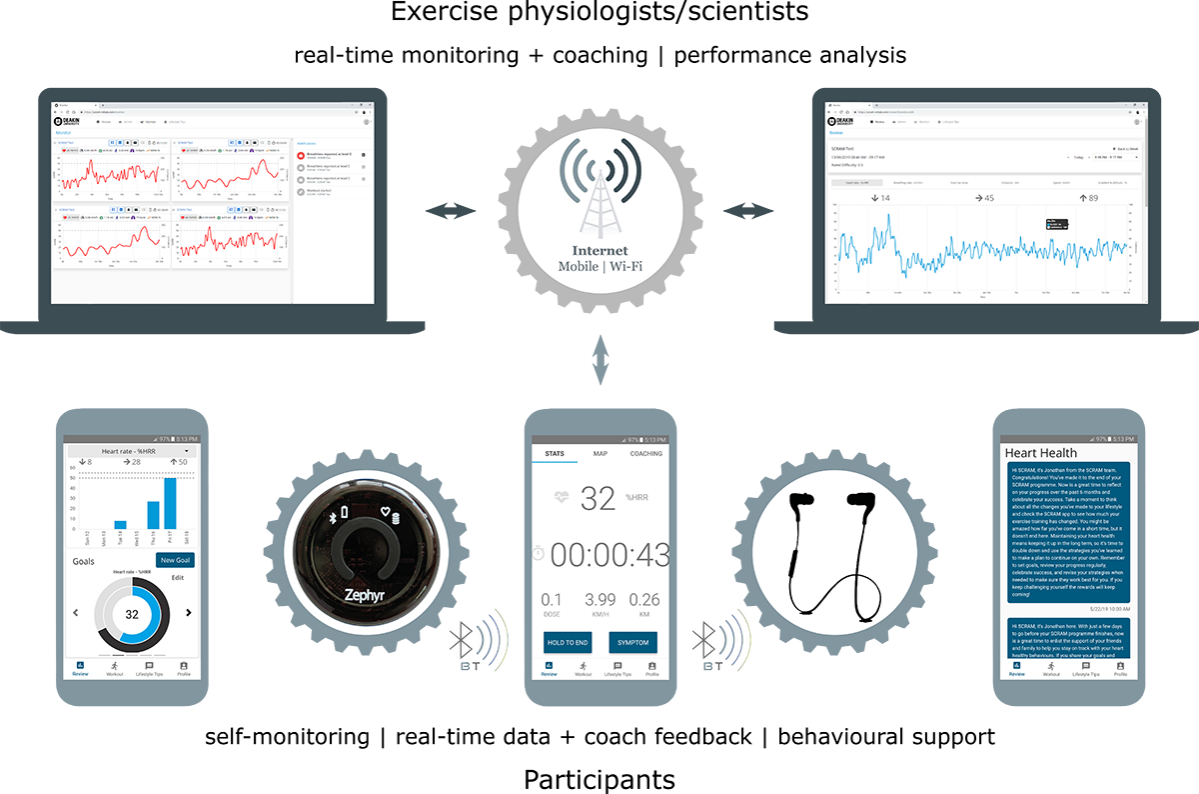
*Smartphone Cardiac Rehabilitation, Assisted self-Management* mobile health platform components.

The bespoke SCRAM mHealth platform builds on our previous research [15] and includes a wearable sensor (BioHarness 3, Zephyr Performance Systems), a participant-facing app that is compatible with the Android mobile operating system (≥v5.0), and a CR professional-facing Web app that is compatible with mobile and desktop Web browsers (Figure 2). Participants access the smartphone app via a Google Play Store beta test program. Both apps have been redeveloped to ensure compatibility with recent updates to the Android mobile operating system, include improvements arising from our previous research [16], and integrate additional behavior support features.

Participants are familiarized with wearable sensor and smartphone app operation during bespoke intervention training immediately after randomization and receive an illustrated user guide. Participants also receive an induction phone call before their first scheduled exercise training session to build rapport with SCRAM exercise physiologists and reinforce operation of the technology platform. Additional technical support is provided as required throughout the trial via email and telephone.

As it is unethical to withhold evidence-based standard care, participants in the intervention arm can also self-initiate access to usual care CR, as described above. It is unclear how many participants will choose to access SCRAM and usual care CR; however, widespread low uptake of center-based CR suggests few will electively complete both programs. Self-reported usual care CR utilization will be assessed to explore the impact on trial outcomes.

### Intensive Phase (Weeks 1-12)

#### Exercise Training

During the initial intensive phase, real-time exercise monitoring and coaching are available during predefined operating hours (3 mornings, 06:00-10:00, plus 2 evenings 17:30-19:30 per week). At preferred times within these periods, participants self-fit the wearable sensor and activate the smartphone app to connect with remotely located exercise physiologists. Physiological (heart and respiratory rates, 1-lead electrocardiogram), geospatial (location, distance, speed—measured via Android location services), and self-reported symptom data (if any) are transmitted to a cloud server and visualized in the Web app for review by exercise physiologists, all in real time. Participants receive real-time individualized coaching, feedback, and support from exercise physiologists—based on exercise performance data—throughout exercise training via smartphone app text-to-voice audio notifications. Outside of predefined operating hours, participants use the smartphone app to self-monitor their exercise performance (in real time during exercise and retrospectively after exercise—using automated graphing elements); set exercise goals and review goal achievement feedback (via automated graphing elements); and receive evidence- and theory-based behavioral support via push notifications (see below).

During operating hours, exercise physiologists use the Web app to monitor participants’ location, distance, speed, heart and respiratory rates, electrocardiogram, and self-reported cardiac symptoms (if any), as well as provide participants with regular real-time coaching instruction, feedback, and support during exercise. Coaching interactions are delivered to participants via a text-to-voice protocol. Exercise physiologists also retrospectively review participants’ exercise performance and goal achievement data via the Web app to inform individualized and progressive exercise prescription, and they may contact participants via telephone and email to discuss program progression if required. Collectively, these features enable optimal individualization and progression of participants’ exercise prescription throughout the 12-week intensive phase.

Exercise physiologists received initial training to apply their expertise via the SCRAM platform, and they receive ad hoc support to maintain intervention fidelity and quality throughout the trial.

The SCRAM platform supports simultaneous monitoring of multiple participants, and the use of mobile and Web apps allows participants and exercise physiologists to operate in any environment with an active Wi-Fi or third/fourth generation cellular broadband connection; data use is typically less than 5 MB per participant, per hour of exercise training [15]. This level of oversight is similar to center-based programs and a significant improvement on existing home-based CR.

Following American College of Sports Medicine guidelines [30], exercise physiologists provide individualized exercise prescription based on participants’ clinical status, exercise capacity, exercise-induced cardiac signs and symptoms (if any), age, sex, and personal preferences. Exercise frequency, duration, and intensity-level targets are delivered to participants via the smartphone app. Initial prescription targets 3 sessions per week of 30 to 40-min duration at 40% to 50% heart rate reserve (HRR). Exercise prescription parameters are progressively increased according to participants’ exercise tolerance, signs and symptoms, and clinical status, targeting 3 to 5 weekly sessions of 60 min at 65% to 85% HRR by the end of the 12-week intensive phase. Prescribed exercise intensity levels are sufficient to induce physiological adaptation without inducing abnormal clinical signs or symptoms. All exercise training sessions include warm-up and cooldown periods to enable appropriate cardiovascular and musculoskeletal preparation and recovery. Walking is the preferred exercise mode, as it does not require specific equipment; however, participants can choose other land-based modes if preferred.

#### Self-Management Behavioral Strategies

Participants receive modular evidence- and theory-based smartphone push notifications that are designed to facilitate uptake and maintenance of chronic disease self-management behaviors. Behavioral content includes information (ie, education) and strategies (ie, behavior change techniques) to support heart health, physical activity and sedentary behavior, healthy eating and alcohol consumption, stress management, and—when indicated—smoking cessation (Table 1). Educational and behavioral content progresses over the intervention period to promote uptake, short-term adherence, relapse prevention/reuptake, and long-term self-determined maintenance. Participants are encouraged to activate all modules to facilitate multifactorial behavioral support, but they can activate/deactivate individual modules via the SCRAM app to meet their individual needs and preferences. Notification frequency depends on the number of active modules (Table 1). Activation (or reactivation) of modules after the start of the intervention period triggers a single-batch receipt of content that was scheduled for delivery before the (re)activation date. For example, if a module is activated 2 weeks after starting the intervention, all messages scheduled for receipt during the first 2 weeks are delivered in a single batch. Subsequent messages are received as per the normal delivery schedule. This approach maximizes behavioral support and preserves the progressive nature of education and behavior content over the intervention period, while also enabling participants to tailor the types of support they receive to meet their individual needs.

**Table 1:** Outline of self-management behavioral support modules.

Notification content			Weekly frequency (n)		
Module	Topics		Intensive phase		Maintenance phase
Heart health	Information about CHD^a^ risk factors, medication beliefs, illness perceptions, and medical checkups; behavioral strategies to facilitate uptake and maintenance.	4		3	
Physical activity and sedentary behavior	Information about the physical activity, sedentary behavior; activity suggestions; behavioral strategies to facilitate uptake and maintenance.	2		1	
Healthy eating	Dietary recommendations for vegetables, fruit, wholegrains, legumes, fat, salt, and alcohol; emphases on variety, recipes, meal planning and preparation methods, cooking techniques, and mindfulness; behavioral strategies to facilitate uptake and maintenance.	2		1	
Stress management	Information about emotional strain and negative affect, as well as strategies to facilitate uptake and maintenance of stress management behaviors.	2		1	
Smoking cessation (if indicated)	Information about the impact of tobacco smoking on health and sources of smoking cessation support, as well as strategies to facilitate the reduction and cessation of tobacco smoking.	2		1	

^a^CHD: coronary heart disease.

### Maintenance Phase (Weeks 13-24)

#### Exercise Training

Participants continue to receive individualized and progressive exercise prescription during the maintenance phase; however, the format of supervision and coaching changes. Participants quantify their exercise performance using the SCRAM smartphone app and wearable sensor as per the intensive phase, but they no longer receive real-time supervision or coaching throughout training sessions. Instead, exercise physiologists provide individualized coaching feedback and update exercise prescription parameters during weekly telephone calls, based on assessment of participants’ exercise performance and goal achievement using retrospective review features of the Web app. Nonadherence (ie, absence of recorded data) initiates prompts from exercise physiologists to promote reengagement.

As per the intensive phase, participants use the smartphone app to self-monitor exercise performance (in real time during exercise and retrospectively afterward using automated graphing elements); set exercise goals and review goal achievement feedback (using automated graphing elements); and receive evidence- and theory-based behavioral support push notifications.

### Self-Management Behavioral Support

Self-management behavioral support—as described above—is delivered at a reduced frequency during the maintenance phase; notification frequency depends on the number of active modules (Table 1). Content emphasizes behavioral strategies that promote long-term adherence, including autonomy, self-directed motivation, and relapse prevention.

#### Theoretical Framework

Features and content of the SCRAM intervention are informed by Social Cognitive Theory, Self-Determination Theory, the Common Sense Model, and the Taxonomy of Behavior Change Techniques [31-34]. Theory-based platform components are designed to enhance task and barrier self-efficacy; perceptions of competence, autonomy, and relatedness; and perceptions of control over health and well-being, as these constructs influence the uptake and adherence of self-management behaviors [35-38].

Real-time biofeedback and exercise coaching during the intensive phase have been designed to support task self-efficacy and perceived competence by providing information about participants’ exercise performance and progress toward prescribed exercise targets (ie, reinforcing mastery experiences via verbal persuasion). Delivery of exercise coaching by real accredited exercise physiologists—rather than automated processes—was chosen to enhance participants’ perceived relatedness by providing a sense of connection and social support.

The remote intervention delivery model has been designed to support participants’ perceived autonomy by enabling them to choose preferred aerobic exercise modes, exercise locations/routes, and exercise times (within predefined operating hours during the intensive phase). Enabling participants to develop exercise task and barrier self-efficacy in their own environment may also support long-term behavior change by overcoming barriers associated with transitioning away from the specialized facilities and equipment provided in center-based settings.

The smartphone app dashboard has been designed to support perceived competence and task self-efficacy by enabling participants to set individualized exercise goals, view automated goal achievement feedback, and self-monitor their exercise performance and progression throughout the intervention. Self-monitoring features provide access to brief summary data, as well as full-resolution data, to cater to varying participant engagement and preferences.

Behavioral support content builds on our previous CR SMS interventions that have been shown to improve self-management behaviors [10,39,40]; the larger character allowance of push notifications has been used to integrate more natural language than is typically possible with a 160-character SMS.

Table 2 summarizes the integration of 24 separate behavior change techniques into the exercise training and behavior change support components of the SCRAM intervention. Two techniques are implicit in the dual-phase design (7.3 Reduce prompts/cues) and inclusion of accredited exercise physiologists for exercise program delivery, as well as behavioral scientists for push notification content creation (9.1 Credible source). The remaining 22 techniques are collectively used 622 times across all push notification modules. Instances of behavior change techniques cannot be prospectively quantified for the exercise monitoring/coaching and goal setting/self-monitoring intervention components, as they are dependent on exercise physiologist interactions and participants’ app usage, respectively.

**Table 2:** Integration of behavior change techniques into *Smartphone Cardiac Rehabilitation, Assisted self-Management* exercise training and behavioral support intervention components.

Behavior change technique	Exercise training		Push notification modules (n)					
	Monitoring+coaching	Goals+self-monitoring	Heart health	Physical activity	Healthy eating	Stress management	Smoking cessation	Total
1.1 Goal setting (behavior)	Yes	Yes	22	9	12	10	6	59
1.2 Problem solving	N/A^a^	N/A	17	18	6	3	10	54
1.4 Action planning	N/A	N/A	31	12	17	15	15	90
1.5 Review behavior goal(s)	Yes	Yes	6	4	0	N/A	4	14
1.6 Discrepancy between current behavior and goal	Yes	Yes	10	5	2	N/A	N/A	17
2.3 Self-monitoring of behavior	Yes	Yes	42	19	24	20	15	120
3.1 Social support (unspecified)	Yes	N/A	7	0	1	2	0	10
3.3 Social support (emotional)	Yes	N/A	15	9	1	9	8	42
4.1 Instruction on how to perform behavior	Yes	N/A	17	16	14	12	3	62
5.1 Information about health consequences	N/A	N/A	9	4	12	9	10	44
7.1 Prompts/cues	N/A	N/A	3	0	0	0	2	5
7.3 Reduce prompts/cues	Yes^c^	N/A	Yes^b^	Yes^b^	Yes^b^	Yes^b^	Yes^b^	Yes^b^
8.2 Behavior substitution	N/A	N/A	2	2	8	0	0	12
8.3 Habit formation	N/A	N/A	3	2	0	0	0	5
8.4 Habit reversal	N/A	N/A	0	1	0	0	1	2
8.7 Graded tasks	Yes	N/A	5	4	3	0	3	15
9.1 Credible source	Yes^c^	N/A	Yes^c^	Yes^c^	Yes^c^	Yes^c^	Yes^c^	Yes^c^
10.4 Social reward	Yes	N/A	16	13	5	2	5	41
10.9 Self-reward	N/A	N/A	2	0	0	0	0	2
11.1 Pharmacological support	N/A	N/A	1	0	0	0	3	4
12.1 Restructuring the physical environment	N/A	N/A	2	0	0	0	1	3
12.3 Avoidance/reducing exposure to cues for the behavior	N/A	N/A	0	0	0	0	3	3
15.1 Verbal persuasion about capability	Yes	N/A	5	1	1	2	3	12
15.3 Focus on past success	Yes	Yes	11	0	1	2	0	14
Number of discrete behavior change technique within intervention components	11	5	20	15	14	11	16	24
Total instances of behavior change technique use within intervention components	—^d^	—^d^	226	119	107	86	92	630

^a^Not applicable.

^b^Implicit in the dual-phase intervention design.

^c^Implicit in inclusion of accredited exercise physiologists and behavioral scientists in intervention design and delivery.

^d^Instances of behavior change technique use cannot be prospectively quantified for these features.

#### Outcomes

Outcome measures and the assessment schedule are summarized in Table 3. 

Baseline and 24-week assessments are conducted at trial centers; 12-week assessments are conducted via telephone and internet survey. Furthermore, 12- and 24-week assessments are permitted ±2 weeks of the scheduled date to meet participant scheduling commitments. Participants who do not attend assessments are rescheduled for a second appointment; those who do not complete the 12-week assessment but do not withdraw their participation are contacted to complete the 24-week assessment. If participants cannot attend the 24-week assessment in person, self-report data may be collected via telephone and internet survey to minimize missing data.

Demographic and eHealth literacy data are collected at baseline to describe sample characteristics. Clinical data (diagnosis and treatment, hospitalization, and medication) are obtained with consent from hospital records, where possible. Self-reported adverse events are recorded and/or updated throughout the participation period as required.

**Table 3:** Schedule of enrollment, treatments, and assessments.

Activity		Description		Weeks −4 to 0		Week 0		Week 12		Week 24	
Enrollment		Contact details, eligibility criteria, verbal consent for trial referral		Yes		N/A^a^		N/A		N/A	
Consent		Trial procedures, health care/medication utilization data release		N/A		Yes		N/A		N/A	
Allocation		Concealed centralized computer-based randomization		N/A		Yes		N/A		N/A	
**Treatments**											
	Control		Usual cardiac rehabilitation care alone		N/A		Start		Ongoing		Finish
	Intervention		*Smartphone Cardiac Rehabilitation, Assisted self-Management*		N/A		Start intensive phase		Start maintenance phase		Finish
**Characteristics**											
	Sociodemographic		Age, sex, ethnicity, income, education, and occupation		N/A		Yes		N/A		N/A
	Clinical		Diagnostic, smoking and alcohol history, and medication		N/A		Yes		N/A		N/A
	Electronic health literacy		Electronic Health Literacy Questionnaire [41]		N/A		Yes		N/A		N/A
**Primary outcome**											
	VO_2_max^b^		Maximal treadmill cardiopulmonary exercise test		N/A		Yes		N/A		Yes
**Secondary outcomes**											
	Anthropometry		Body mass, body mass index, and waist and hip circumferences		N/A		Yes		N/A		Yes
	Blood lipid concentrations^c,d^		Total/high-density lipoprotein/low-density lipoprotein cholesterol, and triglyceride		N/A		Yes		N/A		Yes
	Blood glucose concentration^c,d^		≥3 hours fasted		N/A		Yes		N/A		Yes
	Blood pressure^c,d,e^		Automated sphygmomanometer		N/A		Yes		N/A		Yes
	Physical activity		Godin Leisure Time Physical Activity Questionnaire, % reporting leisure score index ≥ 14 units [42]		N/A		Yes		Yes		Yes
	Dietary intake		Automated self-administered 24-hour dietary assessment tool-Australia, dietary guideline index, and vegetable and discretionary food consumption [43-46]		N/A		Yes		Yes		Yes
	Alcohol consumption		3-item Alcohol Use Disorders Identification Test-C Questionnaire, % reporting ≤2 drinks/day [47]		N/A		Yes		Yes		Yes
	Medication adherence		4-item Medication Adherence Scale, % adherent (score=4) [48]		N/A		Yes		Yes		Yes
	Health-related quality of life		Assessment of Quality of Life 8-dimension, multiattribute utility score [49]		N/A		Yes		Yes		Yes
	Adverse events^f^		Self-reported changes to health status		N/A		Yes		Yes		Yes
Economic evaluation		Delivery, health care/medication use, and participant costs		N/A		N/A		N/A		Yes	
Process evaluation		Semistructured interviews; utilization data		N/A		N/A		N/A		Yes	

^a^Not applicable.

^b^VO_2_max: maximal aerobic exercise capacity.

^c^Measured after ≥3 hour fast.

^d^Measured before cardiopulmonary exercise test.

^e^Measured after ≥10 min of seated rest.

^f^Documented as required throughout the participation period.

#### Primary Outcome

The primary outcome is maximal oxygen uptake (VO_2_max, mL·kg^−1^·min^−1^) measured at 24 weeks. VO_2_max is the criterion measure of maximal cardiorespiratory fitness [30], and an important clinical surrogate outcome as a 1 mL·kg^−1^·min^−1^ improvement has been associated with a 10% reduction in cardiovascular mortality among both men and women [50,51].

After a 5-min warm-up, an individualized treadmill cardiopulmonary exercise test is initiated at a self-selected velocity and 0% gradient. Thereafter, gradient is increased by 1% every 60 seconds until volitional exhaustion or indications for test termination [30]. A 5-min cooldown is completed at or below the self-selected velocity and 0% gradient. Cardiorespiratory responses are measured using a calibrated online metabolic cart; VO_2_max is defined as the mean value across 15 consecutive breaths before fatigue or symptom-limited test termination. Modification of treadmill velocity is permitted during the protocol if physiological responses indicate the self-selected velocity is insufficient to achieve an 8 to 12-min target test duration.

The individualized protocol is replicated at 24-week follow-up assessment; further modification of treadmill velocity is permitted after surpassing the baseline test duration, if required, to achieve a maximal performance.

Test procedures follow clinical guidelines, including continuous monitoring of 12-lead electrocardiogram, frequent assessment of blood pressure and patient-reported cardiovascular signs and symptoms, and medical support to administer treatment in the event of exercise-induced complications [30].

#### Secondary Outcomes

Secondary outcomes are outlined in Table 3. Resting systolic and diastolic blood pressure are measured after ≥10-min seated rest, in duplicate, to the nearest 1 mmHg, using a calibrated automated sphygmomanometer. A third measure is taken if duplicates differ by >10 mmHg; outcomes are recorded as the mean of the closest 2 measures.

Anthropometry outcomes are measured in duplicate with calibrated tools. Stature and body mass are measured to the nearest 0.1 cm and 0.1 kg, respectively. Third measures are taken if duplicates differ by >0.5 cm or >0.2 kg, respectively. Waist and hip circumferences are measured in duplicate to the nearest 0.1 cm at the levels of the umbilicus and the furthest posterior protrusion of the buttocks, respectively. Third measurements are taken if duplicates differ by >0.5 cm; all anthropometric outcomes are recorded as the mean of the closest 2 measures. Body mass index is calculated as weight (kg)/height (m)^2^. Waist-hip ratio is calculated as waist circumference (cm)/hip circumference (cm).

Blood lipid and glucose concentrations are measured via peripheral capillary blood samples using a calibrated point-of-care analyzer (Cholestech LDX, Abbott). Participants are asked to fast (no food or nonwater beverages) for at least 3 hours before the appointments and blood samples are drawn before the cardiopulmonary exercise test.

Self-management behaviors are measured using validated self-report instruments (Table 3), including physical activity (Godin Leisure-time Physical Activity Questionnaire) [42], dietary intake (automated self-administered 24-hour dietary assessment tool [ASA24]-Australia) [43-46], alcohol consumption (Alcohol Use Disorders Identification Test-C) [47], medication adherence (Medication Adherence Scale) [48], and health-related quality of life (Assessment of Quality of Life 8-dimension) [49]. The ASA24-Australia is a Web-based diet recall tool. Participants complete a Web-based recall for 1 calendar weekday within 3 days of assessment appointments; those who require help complete the recall with a researcher at assessment appointments or via telephone. Recalls are audited to identify possible misreporting and participants are asked to complete a new recall if misreporting is confirmed.

#### Economic Evaluation

Detailed methods for the economic evaluation will be described separately but, briefly, the evaluation will adopt a health care system and a limited societal perspective incorporating all health care costs subsidized by state and Commonwealth governments in Australia. Types and costs of inpatient and outpatient health care as well as dispensed medications will be estimated from self-reported adverse events, the Australian Government Medicare Benefits Schedule, and the Australian Government Pharmaceutical Benefits Schedule, respectively. Productivity loss will be measured by self-reported work absenteeism.

A preference index for the calculation of quality-adjusted life years (QALYs) will be derived from the 8-dimension Assessment of Quality of Life to assess cost per QALY for comparison with other programs. Incremental costs per unit increase in the primary (VO_2_max) and secondary outcomes will also be calculated. Extensive probabilistic uncertainty analysis will be undertaken in addition to the base case analysis.

#### Process Evaluation

Semistructured exit interviews are conducted to understand usability, acceptability, engagement, and satisfaction with the SCRAM and usual care CR treatments, respectively. SCRAM treatment fidelity is assessed via exercise session completion (determined from recorded exercise training data), self-reported compliance with behavioral notifications, and SCRAM platform usage metrics. Usual care treatment fidelity is assessed via self-reported uptake of standard CR services. Process data will be summarized using descriptive statistics (quantitative questions and analytics) and general inductive thematic analyses (open-ended questions). Interview data will be transcribed verbatim and analyzed using a general inductive thematic approach [52].

### Data Collection and Analysis

#### Data Collection and Management

Primary outcome assessors have previous experience with cardiopulmonary exercise testing, and all outcome assessors completed standardized training for required data collection protocols/methods to ensure consistency across all 3 trial centers.

Trial data are collected and managed using REDCap tools hosted at Deakin University [53,54]. REDCap is a secure, Web-based software platform designed to support data capture for research studies. Trial data are recorded directly into REDCap case record forms or, if required, on hard copy case record forms and transcribed into REDCap as soon as possible. Data entry is conducted by outcome assessors to maintain blinding. Validation rules (eg, range and completion checks) are prespecified in REDCap to identify inaccuracies and missing data; outcome assessors respond to queries as required. Hard copy corrections (if required) are initialed and dated by outcome assessors and transcribed into REDCap as soon as possible; REDCap data logging maintains an auditable record of all electronic data entries and corrections.

Electronic data are stored on a secure password-protected server; hard copy data are stored in secure filing cabinets at trial centers until they can be transferred to the coordinating trial center. Access to trial data is restricted to members of the research team. A data manager and individuals approved by the trial steering group retain access to data from all trial centers; however, outcome assessors can only access data for their individual trial center. Unique reidentifiable codes are used to anonymize trial data. Access to identifiable data is permitted for the purposes of contacting participants, verifying data entry, analysis, auditing, or other purposes approved by the Steering Group. There are no significant or uncertain risks of harm associated with the SCRAM intervention or usual care CR, and no interim analyses are planned. Accordingly, no data safety monitoring board has been established [55].

#### Statistical Analysis

Baseline characteristics will be summarized descriptively for the full sample and by treatment group. A linear regression model—adjusted for baseline values and stratification variables—will be fitted to assess the primary outcome. Primary analyses will be performed on the principle of intention to treat. Assuming there is a reasonable amount (>5%) of missing data, primary analyses will be conducted using multiple imputation, imputing all outcomes using a single (joint) imputation model. Imputation models will include participant age, sex, type of diagnosis (eg, angina, myocardial infarction, and coronary revascularization), in addition to any other covariates that appear to predict missingness. A per-protocol analysis will be conducted with complete cases to test the robustness of treatment effects under different assumptions. Preplanned subgroup analyses will be conducted to determine treatment effects by levels of treatment adherence, sex, and trial center (Figure 1). Secondary analyses will adjust the regression model for baseline prognostic factors, including age, sex, and employment status.

A similar approach will be used to assess continuous secondary outcomes (Table 3); logistic regression will be used for binary outcomes. Model-adjusted estimates and group differences will be reported with 95% CIs and probability values. All statistical tests will be 2-sided at α=.05. The biostatistician will remain blinded throughout the analysis of treatment effects. A detailed statistical analysis plan will be developed before the completion of data collection to guide analyses.

#### Sample Size

Our previous noninferiority trial [10] indicates that 174 participants are required to detect a clinically meaningful between-group difference of 2.0 mL**·**kg^−1^**·**min^−1^ in VO_2_max (SD 6.75 mL**·**kg^−1^**·**min^−1^, r=0.80 between repeated measures, β=.9, and 2-sided α=.05). Allowing for 20% loss to follow-up (17% in our previous trial), a total of 220 participants are required to ensure equal numbers per treatment arm (ie, 110 per arm). Minimum detectable between-group differences in secondary outcomes at 24-week follow-up are summarized in Table 4.

**Table 4:** Minimum detectable differences in secondary outcomes (assuming a total sample size of 220 participants [110 per treatment arm] and beta=.8).

Secondary outcome	Group difference	Data source
Total cholesterol concentration	0.24 mmol·L^−1^	Maddison et al [10]
Low-density lipoprotein cholesterol concentration	0.16 mmol·L^−1^	Maddison et al [10]
High-density lipoprotein cholesterol concentration	0.10 mmol·L^−1^	Maddison et al [10]
Triglyceride concentration	0.29 mmol·L^−1^	Maddison et al [10]
Glucose concentration	0.60 mmol·L^−1^	Maddison et al [10]
Physical activity leisure score index	17%	Dale et al [40]
Health-related quality of life	0.06 units	Richardson et al [56]
Medication adherence	19%	Thornley et al [57]
Systolic blood pressure	4.70 mmHg	Maddison et al [10]
Diastolic blood pressure	2.76 mmHg	Maddison et al [10]
Body mass	3.80 kg	Maddison et al [10]
Body mass Index	0.90 kg/m^2^	Maddison et al [10]
Waist circumference	2.60 cm	Maddison et al [10]
Hip circumference	1.80 cm	Maddison et al [10]
Alcohol consumption	21%	Dale et al [40]
Dietary guideline index	3.70 units	Livingstone and McNaughton [45]

### Protocol Amendments

The medication adherence self-report instrument was changed to the 4-item Morisky, Green, and Levine Medication Adherence Scale [48]—before prospective clinical trial registration—to comply with trademark restrictions on the 8-item Morisky Medication Adherence Scale.

Amendments will be formally documented in the version-controlled trial protocol. Changes will be disseminated to the approving ethics committees and participants as required, and the trial registration will be updated as required.

### Availability of Data

Following trial completion and publication, requests for deidentified individual participant data or trial documents will be considered where the proposed use complies with trial ethical approval, does not conflict with planned use by the trial steering committee or other external data requests, and aligns with public good purposes, as well as where the requestor is willing to sign a data access agreement.

### Dissemination

The trial will be disseminated via scientific conferences, peer-reviewed scientific journals, and other channels approved by the steering group. No identifiable data will be included in any outputs. Authorship for trial publications will be determined in accordance with International Committee of Medical Journal Editors authorship recommendations [58].

### Trial Status

This manuscript describes version 2 (11/30/2018) of the full trial protocol. Recruitment began on November 12, 2018, and is anticipated to be completed by March 31, 2020.

### Ethics Approval and Consent to Participate

The trial protocol has been approved under Australia’s National Mutual Acceptance agreement by the Melbourne Health Human Research Ethics Committee (HREC/18/MH/119). Ethical approval has been ratified by the Deakin University Human Research Ethics Committee (2018-251). All participants provide written informed consent before undertaking baseline assessments. Separate consent is sought to extract health care and medication utilization data for the purpose of this trial and for participation in substudies, as required.

## Results

The trial was funded in November 2017 and received ethical approval in June 2018, and recruitment began in November 2018. As of September 2019, 54 participants have been randomized into the trial.

## Discussion

CR delivery has remained largely unchanged for several decades, and despite proven effectiveness [12,59], persistent low participation rates mean its potential to improve individual, clinical, and economic outcomes has not been fully realized. Accessibility barriers are important limitations of current CR delivery, and overcoming those challenges is a priority as people with lower access to center-based CR have more adverse risk factor profiles [60].

This trial is among the first to evaluate an evidence- and theory-based multiphase mHealth CR intervention that can deliver real-time exercise supervision plus modular self-management support to participants in almost any geographic location. SCRAM combines key elements of our previous research into a single mHealth platform to address key accessibility barriers while preserving individualized oversight from CR professionals. As mobile broadband services cover 95% of the Australian population, the near-universal accessibility of the SCRAM program may (1) help to reduce geographic and socioeconomic inequalities in CR utilization, particularly in regional and rural areas where accessibility barriers are exacerbated [17], and (2) facilitate large-scale implementation in clinical practice.

Trial outcomes are associated with cardiac and all-cause mortality, and the inclusion of a geographically diverse cohort (metropolitan-, regional-, and rural-dwelling participants) will help to understand the role of mHealth CR across multiple health care settings with differing needs.

Our trial will also provide unique and valuable information about costs and cost-effectiveness, which are critical to inform health care providers about the potential for SCRAM to be implemented at scale in clinical practice—as a complement to existing CR services. Finally, direct involvement of clinicians and CR professionals in this research improves the prospect for timely translation of findings into clinical practice if the intervention is proven to be effective and cost-effective.

## Appendix

Multimedia Appendix 1 Standard Protocol Items: Recommendations for Interventional Trials Checklist (2013).

Multimedia Appendix 2 Template for Intervention Description and Replication Checklist.

Multimedia Appendix 3 Consolidated Standards of Reporting Trials Checklist–electronic health extension.

## Abbreviations

ASA24: automated self-administered 24-hour dietary assessment tool
CHD: coronary heart disease
CONSORT: Consolidated Standards of Reporting Trials
CR: cardiac rehabilitation
eHealth: electronic health
HRR: heart rate reserve
mHealth: mobile health
QALY: quality-adjusted life year
RCT: randomized controlled trial
REDCap: Research Electronic Data Capture
REMOTE-CR: Remote Exercise MOnitoring Trial for Exercise-based Cardiac Rehabilitation
SCRAM: *Smartphone Cardiac Rehabilitation, Assisted self-Management*
