# Supplementary material for: Smartphone Cardiac Rehabilitation, Assisted Self-Management Versus Usual Care: Protocol for a Multicenter Randomized Controlled Trial to Compare Effects and Costs Among People With Coronary Heart Disease
Source: JMIR Res Protoc. 2020 Jan 27;9(1):e15022. doi: 10.2196/15022 (PMC7011127; doi:10.2196/15022)
Supplement: Multimedia Appendix 3 [file resprot_v9i1e15022_app3.docx]

Appendix 3. Consolidated Standards of Reporting Trials Checklist–eHealth extension

| **Section** | **Item** | **Description** | **page** |
| --- | --- | --- | --- |
| **Title & Abstract** | 1a | Identification as a randomised trial in the title | 1 |
|  | 1b | Structured summary of trial design, methods, results, and conclusions | 2 |
| **Introduction** |  |  |  |
| Background | 2a | Scientific background and explanation of rationale | 4 |
|  | 2b | Specific objectives or hypotheses | - |
| **Methods** |  |  |  |
| Trial design | 3a | Description of trial design including allocation ratio | - |
|  | 3b | Important changes to methods after trial commencement (such as eligibility criteria), with reasons | N/A |
| Participants | 4a | Eligibility criteria for participants | 7 |
|  | 4b | Settings and locations where the data were collected | 14 |
| Interventions | 5 | The interventions for each group with sufficient details to allow replication, including how and when they were actually administered | 8 |
| Outcomes | 6a | Completely defined pre-specified primary and secondary outcome measures, including how and when they were assessed | 18 |
|  | 6b | Any changes to trial outcomes after the trial commenced, with reasons | - |
| Sample size | 7a | How sample size was determined | 19 |
|  | 7b | When applicable, explanation of any interim analyses and stopping guidelines | - |
| **Randomisation** |  |  |  |
| Sequence generation | 8a | Method used to generate the random allocation sequence | - |
|  | 8b | Type of randomisation; details of any restriction (such as blocking and block size) | - |
| Allocation concealment | 9 | Mechanism used to implement the random allocation sequence (such as sequentially numbered containers), describing any steps taken to conceal the sequence until interventions were assigned | - |
| Implementation | 10 | Who generated the random allocation sequence, who enrolled participants, and who assigned participants to interventions | - |
| Blinding | 11a | If done, who was blinded after assignment to interventions (for example, participants, care providers, those assessing outcomes) and how | 8 |
|  | 11b | If relevant, description of the similarity of interventions | - |
| Statistical methods | 12a | Statistical methods used to compare groups for primary and secondary outcomes | 19 |
|  | 12b | Methods for additional analyses, such as subgroup analyses and adjusted analyses | - |
| **Results** |  |  |  |
| Participant flow | 13a | For each group, the numbers of participants who were randomly assigned, received intended treatment, and were analysed for the primary outcome | - |
|  | 13b | For each group, losses and exclusions after randomisation, together with reasons | N/A |
| Recruitment | 14a | Dates defining the periods of recruitment and follow-up | N/A |
|  | 14b | Why the trial ended or was stopped | - |
| Baseline data | 15 | A table showing baseline demographic and clinical characteristics for each group | N/A |
| Number analysed | 16 | For each group, number of participants (denominator) included in each analysis and whether the analysis was by original assigned groups | N/A |
| Outcomes & estimation | 17a | For each primary and secondary outcome, results for each group, and the estimated effect size and its precision (such as 95% CI) | N/A |
|  | 17b | For binary outcomes, presentation of both absolute and relative effect sizes is recommended | - |
| Ancillary analyses | 18 | Results of any other analyses performed, including subgroup analyses and adjusted analyses, distinguishing pre-specified from exploratory | N/A |
| Harms | 19 | All important harms or unintended effects in each group | N/A |
| **Discussion** |  |  |  |
| Limitations | 20 | Trial limitations, addressing sources of potential bias, imprecision, and, if relevant, multiplicity of analyses | N/A |
| Generalisability | 21 | Generalisability (external validity, applicability) of the trial findings | 20 |
| Interpretation | 22 | Interpretation consistent with results, balancing benefits and harms, and considering other relevant evidence | 20 |
| **Other** |  |  |  |
| Registration | 23 | Registration number and name of trial registry | - |
| Protocol | 24 | Where the full trial protocol can be accessed, if available | - |
| Funding | 25 | Sources of funding and other support (such as supply of drugs), role of funders | - |

N/A, not applicable to this protocol. -, no additional requirement specified in the eHealth extension.
